# Combined Fuzzy and Genetic Algorithm for the Optimisation of Hybrid Composite-Polymer Joints Obtained by Two-Step Laser Joining Process

**DOI:** 10.3390/ma13020283

**Published:** 2020-01-08

**Authors:** Gennaro Salvatore Ponticelli, Francesco Lambiase, Claudio Leone, Silvio Genna

**Affiliations:** 1 Department of Enterprise Engineering, University of Rome Tor Vergata, via del Politecnico 1, 00133 Rome, Italy; 2 Department of Engineering, University Niccolò Cusano, via Don Carlo Gnocchi 3, 00166 Rome, Italy; 3 Department of Industrial and Information Engineering and Economics, University of L’Aquila, via G. Gronchi, 67100 L’Aquila, Italy; francesco.lambiase@univaq.it; 4 CIRTIBS Research Centre, University of Naples Federico II, P.le Tecchio 80, 80125 Naples, Italy; claudio.leone@unicampania.it; 5 Department of Engineering, University of Campania, via Roma 29, 81031 Aversa (CE), Italy

**Keywords:** laser cleaning, laser joining, hybrid joints, fuzzy logic, genetic algorithms, CFRP.

## Abstract

In the present work, genetic algorithms and fuzzy logic were combined to model and optimise the shear strength of hybrid composite-polymer joints obtained by two step laser joining process. The first step of the process consists of a surface treatment (cleaning) of the carbon fibre-reinforced polymer (CFRP) laminate, by way of a 30 W nanosecond laser. This phase allows removing the first matrix layer from the CFRP and was performed under fixed process parameters condition. In the second step, a diode laser was adopted to join the CFRP to polycarbonate (PC) sheet by laser-assisted direct joining (LADJ). The experimentation was performed adopting an experimental plan developed according to the design of experiment (DOE) methodology, changing the laser power and the laser energy. In order to verify the cleaning effect, untreated laminated were also joined and tested adopting the same process conditions. Analysis of variance (ANOVA) was adopted to detect the statistical influence of the process parameters. Results showed that both the laser treatment and the process parameters strongly influence the joint performances. Then, an uncertain model based on the combination of fuzzy logic and genetic algorithms was developed and adopted to find the best process parameters’ set able to give the maximum joint strength against the lowest uncertainty level. This type of approach is especially useful to provide information about how much the precision of the model and the process varies by changing the process parameters.

## 1. Introduction

During the last decade, traditional manufacturing processes have dealt with an increasing demand from the modern industry in terms of processing of new materials and complexity of final products. This is giving rise to new technological frontiers, which can only be overcome with new and advanced production technologies and systems.

The main reason of this revolution must be sought in the request of more and more reduced environmental impact, with low fuel consumption, CO_2_ emissions and at the same time, improved performances for commercial, industrial and military applications [1,2]. In particular, the transport industry is paying great attention to high-performance lightweight hybrid structures for their capability in offering different opportunities that are not achievable if the materials are used individually [3,4], e.g., obtaining components characterized by high strength and toughness, with the presence of transparent areas for inspection or aesthetical reasons. Among the different materials, carbon fibre-reinforced polymers are very well suited for reducing the overall weight of the structure and therefore improving fuel efficiency [5]. However, often it is not possible to directly manufacture the final component because of its dimension or complexity. Therefore, the only available solution is to join smaller components with, in most cases, supports made by different materials, i.e., metals as well as polymers.

When dealing with the joining of different materials, the choice of the best process is not a straightforward task. The joint can be obtained by using an external fastener, as it happens for the mechanical fastening, but this increases the structure weight, costs and processing time because of the realization of the holes [6]. Moreover, the stress concentrations developing around the joints are particularly severe because the load is only transferred on a fraction of the joint area [7], or else by using adhesives. In this case, even if it is possible to achieve an almost uniform distribution of stress, the process requires specialised workers and the preparation of the substrate with long curing time, increasing both production time and costs [4]. Furthermore, the adhesive bonding involves high environmental impact and suffers from environmental sensitivity, i.e., humidity and temperature, with greater uncertainty regarding the long-term structural integrity [8,9]. For these reasons, there has been the necessity to search for a novel and/or improved joining process, with the aim of overcoming or at least limiting the typical issues of the conventional ones.

So far, aside from mechanical joining and adhesive bonding, new combined thermo-mechanical processes, i.e., friction-based processes, have been developed to produce hybrid joints on different materials, i.e., metal-to-polymer [10,11,12] as well as metal-to-composite [13,14] and composite-to-polymer [15,16,17]. However, these processes based on the friction phenomena produce poor finishing surface in the weld region and they usually require very stiff clamping systems because of the high loads involved during the processes. Moreover, an important issue is related to the tool wear, especially in preparing high temperature melting point materials. Special tools can be used, but they are characterized by high cost and low fracture toughness limiting the applications [15].

Besides these, laser-based joining processes appears to be a valid alternative solution for joining different materials, thanks to their ability in providing an instantaneous bonding, a highly localized heating, no vibration, low residual stresses, flatness of the external surfaces, an improved joint aesthetics, all in one step [18,19,20,21]. Moreover, this non-contact thermal-based joining process has a better environmental adaptability and a more uniform stress distribution if compared with the joining methods described above.

Recently, several works have been carried out on the application of the laser joining technology in order to obtain hybrid structures, specifically aimed at identifying the best process parameters’ combination able to guarantee the highest mechanical performances in terms of shear strength and therefore joint durability. The common result is that the joining strength can be improved by pre-treating the surface with different methods. Jiao et al. [22] reports on the realization of a microtextured interface between aluminium and carbon fibre-reinforced thermosetting polymers by means of a machining process after adding a polyamide-based interlayer, obtaining a maximum joining strength about 260% of that without any surface treating. Engelmann et al. [19] found a dependency of the shear properties of the hybrid joint on the structure distances and orientations. In particular, they achieved an increase of shear strength with a bigger undercut groove and a higher structure depth. Lambiase et al. [4] applied the laser sculpturing on the aluminium substrate before joining. This pre-treatment allowed the obtainment of teeth features that improved the joining strength between the aluminium and polycarbonate because of the greater surface available for the joint.

In this context, research in laser joining processes development, optimisation and modelling/simulation plays a critical role in advancing materials joining science and technology. However, the decisions to be made to set-up the process are complex, since a wide range of alternative options must be evaluated, and the choice of the best one is frequently made on a set of conflicting criteria [23]. Therefore, in order to enhance the decision-making process, one can perform experiments and/or develop models. In the first case, process parameters are usually adjusted and tuned one by one to provide the desired requirements [20,24], but this involves lots of resources, and on the same time, final quality cannot be easily predicted. In this light, modelling appears to be very helpful and surely the only solution in order to speed up the characterisation and optimisation of the processes [25,26,27]. In particular, the aim should be the development of a physical model able to simulate in a very precise way the entire process. However, in most cases, this is very difficult because of the strong dynamic nature of the process itself. For this reason, empirical modelling can be considered a valuable solution, and very often the only available tool for researchers and manufacturers which allow to predict and control the final quality of a process. It is important to state here that these empirical models exist only thanks to the experiments and are valid only within the space that is tested. Nevertheless, the results provided by such models are affected by two sources of uncertainty, i.e., related to the process variability and to the simplification introduced by the model itself. The former source of uncertainty is usually random and easily modelled with stochastic methods, while, the latter is a systematic error for which statistics does not provide a useful tool, while fuzzy arithmetic can be used fruitfully [28,29,30,31,32].

In this light, the present work is aimed at proposing a combined fuzzy genetic algorithms procedure for the optimisation of the tensile strength of hybrid single lap joints, made of carbon fibre-reinforced polymer (CFRP) laminates and polycarbonate (PC) sheets, obtained by a two-step laser joining process. Where the first step is the laser pre-treatment of the CFRP (laser cleaning), while the second step is the laser joining between the CFRP laminates and PC sheet. At this end, single lap joints made of CFRP and PC were produced changing the average power and the energy released per scan line (by changing the scan speed) on untreated and laser cleaned CFRP laminate. The pre-treatment was carried out on CFRP sheet by using a 30 W Q-switched Yb:YAG fibre laser, while the joining process was performed by using a 200 W nominal power diode laser. Then, a fuzzy-genetic algorithm optimisation model was developed and successfully applied in order to find the best process parameters’ combination able to guarantee the highest mechanical performances with the smallest level of uncertainty, providing as additional information how much the uncertainty of the model and of the process varies by changing the process parameters themselves.

## 2. Materials and Methods

The materials to join are polycarbonate sheets and CFRP laminates. The first was supplied by Bayer and is characterized by a thickness of 2 mm. While, the CFRP plate was manufactured by hot pressing carbon fibre prepregs (0/90°, 50% in the warp and weft directions, MRC Pyrofil, Tokyo, Japan, TR30S and a thermosetting epoxy resin, bisphenol-A type epoxy + phenol Novolac type epoxy) for 2 h at 130 °C and 5 MPa, according to [33,34], obtaining a final thickness of 1.5 mm. The thermal and mechanical characteristics of the materials are summarized in Table 1.

**Figure 1:**
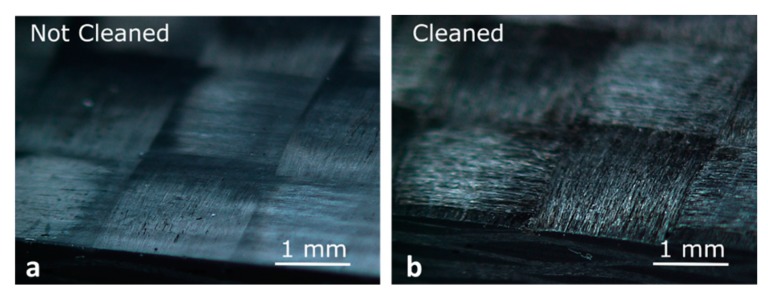
Carbon fibre-reinforced polymer (CFRP) surface (**a**) without and (**b**) with the laser cleaning pre-treatment.

In order to evaluate the effect of the laser cleaning on the mechanical strength of the joints, the removal of the first epoxy matrix layer from the CFRP laminate was performed by using a 30-W fibre laser (YLPRA30-1-50-20-20 by IPG, Oxford, MA, USA), characterised by a focused spot diameter of about 80 µm. The processing conditions are listed in Table 2, while Figure 1 shows the CFRP laminate surface in both the analysed conditions, i.e., with and without the laser cleaning pre-treatment. Such conditions were chosen according to previous experience [35,36].

After cleaning the surface of the CFRP laminate, the laser joining process was performed by using a diode laser with an average power of 200 W (DLR-200-AC, by IPG, Oxford, MA, USA), whose characteristics are reported in Table 3.

**Figure 2:**
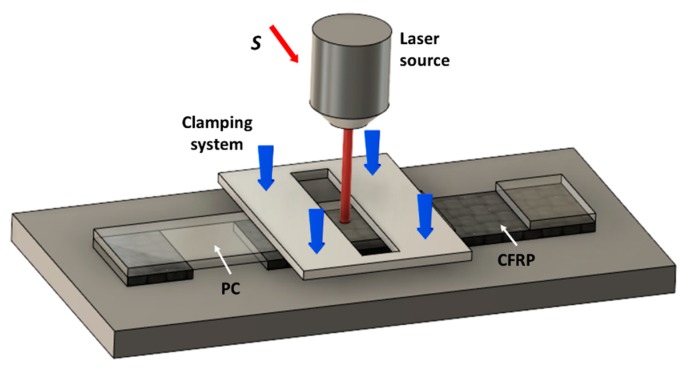
Schematic representation of the joining setup and shear test specimen.

In Figure 2 the schematic of the process is reported: the laser beam is moved on the PC sheet placed on the CFRP laminate. Since the PC is almost transparent to the diode laser radiation [21], the effect of the laser irradiation is the CFRP heating. Thus, the PC is heated and softened by way of the heat absorbed by the CFRP and transferred to it by conduction. In order to ensure the contact between the two materials, a clamping pressure of about 1.0 MPa was applied by way of a clamping frame.

Since a large number of parameters are involved in the laser joining process, the experimental tests were scheduled according to the developed multilevel factorial plan based on design of experiment [37], which is reported in Table 4. In particular, 3 levels of laser power and 3 levels of laser energy were adopted, with and without laser cleaning, for a total of 18 experimental scenarios. Each of them was replicated three times, for a total of 54 tests. It is important to introduce here that, in order to keep constant the laser energy for different values of the laser power, a different laser scan speed has been adopted according to the following equation:

(1) E=PS,

where, *E* is the laser energy per scan line, *P* is the laser power and *S* is the laser scan speed.

**Figure 3:**
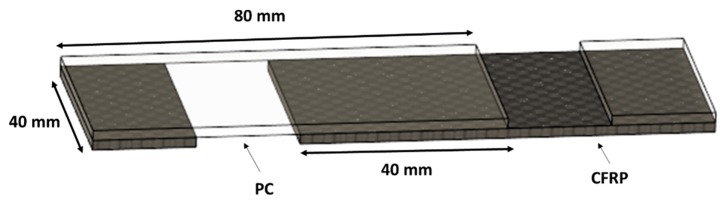
Specimen used for the mechanical characterization.

Mechanical characterization was carried out on single lap joint according to the Figure 3. A universal test machine (model 322.31 by MTS, Eden Prairie, MN, USA) under quasi-static conditions (constant speed of 2 mm/min) was adopted. Ultimate tensile strength (UTS) was calculated as the maximum of the load measured during the test. Finally, the statistical significance of the control factors for the response variable was evaluated by using the analysis of variance (ANOVA) test.

### Computational

Empirical models, exclusively built on the basis of the experimental findings, establish a relationship between input(s) and output(s) making the estimation and the control of the final quality of the process possible. Generally speaking, the adopted relationship is an n-order equation that is optimised to the problem by founding the coefficient and power able to give the minimum error.

However, there is a very high number of feasible relationships (i.e., equations) and the choice of the best one is a challenging and not straightforward task. In addition, the empirical models do not give any information about the process variability. Consequently, also the optimum process conditions obtained by the model are affected by the same problem. In the proposed procedure, the aim of the genetic algorithm is to find the best empirical model while the fuzzification is adopted in order to estimate the uncertainty due to both the process variability and the simplification introduced by the empirical model.

**Figure 4:**
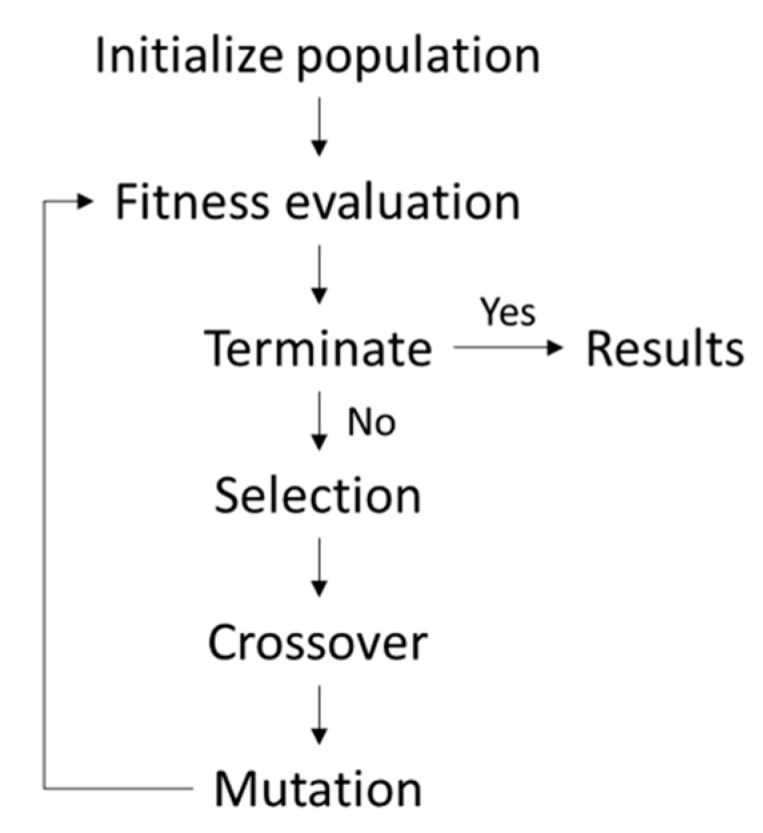
Genetic algorithm procedure.

The general procedure of a genetic algorithm consists of four steps, i.e., initialization, selection, crossover and mutation [38], as shown in Figure 4.

Furthermore, two other important aspects are the definition of the genetic coding and the formulation of the fitness function. In particular, the latter is aimed at evaluating how much the regression model results differ from the experimental data (see Equation (2)).

(2) fit value 1=rms(res_model·k − res_exp),

where, rms represents the operation of root mean square, *res_model* is the result obtained applying the optimal regression model found with the genetic algorithm, *k* is the coefficient of the suggested regression model, evaluated by a multiple linear regression and *res_exp* is the result of the experiments. Specifically, the aim of this part of the model is to minimize the fitness function, therefore finding the best combination of input parameters and their powers.

The initial population of models is generated by assigning to each gene, i.e., each power, a random value within the chosen range. Then, it evolves into the next generation through the genetic operators, i.e., crossover, mutation and selection. The algorithm is then iterated until a defined number of generations in which the fitness value is stationary is reached. It is important to introduce here that the genetic algorithm was used also to find the optimal support of the fuzzy numbers [29] as described hereafter.

**Figure 5:**
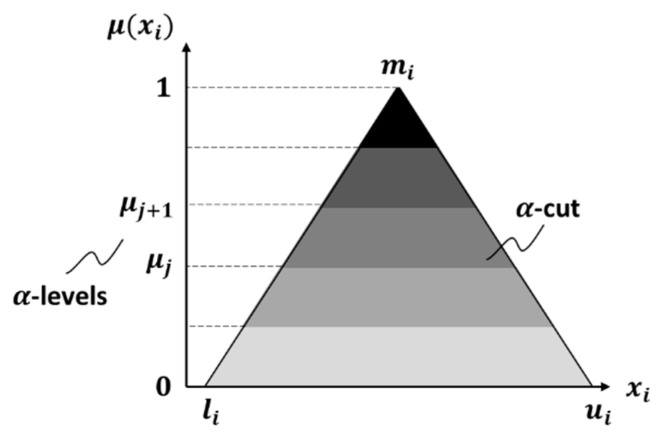
Triangular fuzzy number.

Once the optimal regression model is found, the coefficients are converted in triangular fuzzy numbers, as shown in Figure 5. The reason for choosing these fuzzy numbers is the need of defining an interval for pairwise comparisons, with a lower and upper bound. Within this interval, there should be only one value that is the most likely value for the specific comparison. This definition therefore leads to a triangular fuzzy number. In any case, if there is no reason to suggest otherwise, the shape of the membership function may be assumed to be triangular because of its simplicity in formulation and ease of computation [39].

A triangular fuzzy number is characterized by three values: a lower bound (*l_i_*), an upper bound (*u_i_*) and a modal value (*m_i_*). The modal value has a membership function of 1, the highest possible set membership for uncertain parameters. When the value of the parameter reaches the lower bound (or upper bound) the degree of belief that this value truly represents the chosen parameter is reduced to zero. The interval (*l_i_*, *u_i_*) represents the support of the membership function. Each of the fuzzy regression coefficients has the modal value coinciding with the results provided by the linear regression, while the support is defined by the genetic algorithm as follows:

(3) $$fit\;value\;2=w\cdot \frac{n}{N}+\left(1-w\right)\cdot \frac{H_{V}}{H_{C}}.$$

In Equation (3), *w* is a weighting term, *n* is the number of data not considered, *N* is the total number of data, *H_V_* is the hypervolume covered by the fuzzy results and related to the uncertainty dispersion of the considered data and *H_C_* is the hypercube which covers all the experimental data. It is worth to note that each term is opportunely normalised in order to have two comparable quantities. The aim of such a fitness function is to make the model able to consider the highest number of experimental data in combination with the lowest hypervolume and therefore with the lowest uncertainty level. Also in this case, the convergence of the algorithm is obtained when a fixed number of generations are characterised by the same fitness value.

In order to compute the fuzzy function, the fuzzy number is discretized in a defined number of intervals assigned to the specific α-levels of membership that results from subdividing the possible range of membership by the α-cuts, equally spaced between each other [28], as shown in Figure 5.

In general, the degree of belief behaves according to the following equation:

(4) $$\mu \left(x_{i}\right)=\{\begin{array}{cc} 0, & x_{i}\langle l_{i},\;x_{i}\rangle m_{i}\\ \frac{x_{i}-l_{i}}{m_{i}-l_{i}}, & l_{i}<x_{i}<m_{i}\\ \frac{u_{i}-x_{i}}{u_{i}-m_{i}}, & m_{i}<x_{i}<u_{i} \end{array}.$$

**Figure 6:**
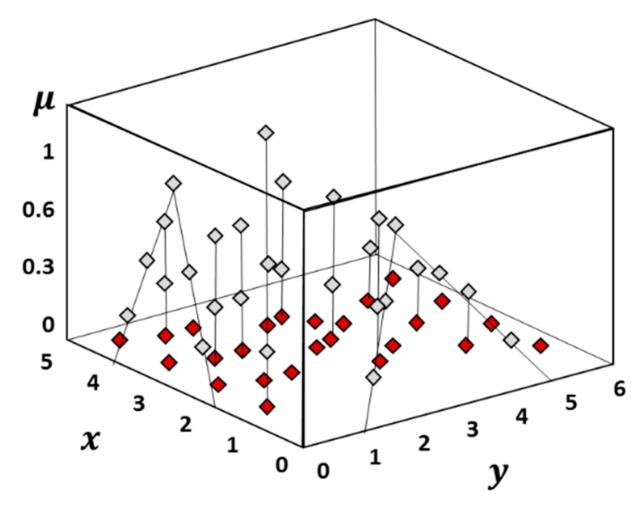
Combinatorial approach of the Transformation Method for two input parameters (x,y).

Finally, the evaluation of the fuzzy function is obtained by using the transformation method, which is a practical implementation of the fuzzy arithmetic [40]. In other words, this method provides the combinatorial scheme of the lower and upper bounds, and/or of additional values in-between, to be adopted for all the uncertain parameters (see Figure 6). This is achieved by assigning a well-structured array to each interval of a fuzzy parameter, which can be considered as a transformation of the interval into a domain where the regular arithmetic for crisp numbers can be applied [41].

## 3. Results and Discussion

**Figure 7:**
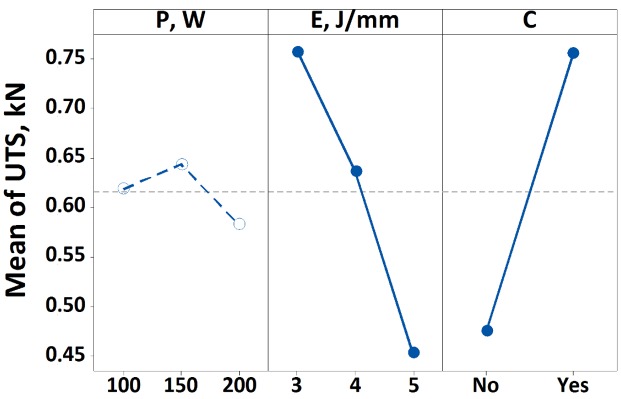
Main effects plot for the ultimate tensile strength.

**Figure 8:**
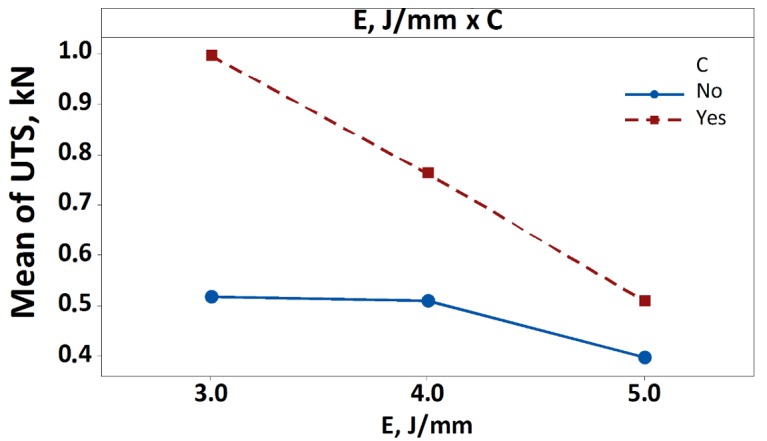
Interaction plot (*E* × *C*) for ultimate tensile strength (UTS).

The experimental results were analysed by means of the ANOVA test, which provides the statistical significance of the control factors (*P*, *E* and *C*), for the ultimate tensile strength (UTS) as response variable. The results consist of a table containing the degrees of freedom (DoF), the sequential sums of squares (Seq.SS), the contribution percentage (Π%) the adjusted sum of squares (Adj.SS), the adjusted mean squares (Adj.MS), the F-value and the *p*-value of each parameter or parameter combination. In general, the term Seq.SS provides a measure of the variation of each parameter with respect to the response variables. This information is quantified by the Π% term, which is the ratio between the Seq.SS term of the analysed parameter and the total one. Unlike the Adj.SS term, the Seq.SS depends on the order the terms are entered into the model. The F-value is used to determine whether a term is associated with the response, comparing the result with the corresponding tabulated value (4.06 for 1-DoF, 3.21 for 2-DoF and 2.59 for 4-DoF): the greater the F-value the greater the influence on the response variable. In this case, the F-value is defined as the ratio between the Adj.MS value of the response variable investigated and the Adj.MS of the error. Finally, the *p*-value is used to determine the significance of the factors (the analysis was carried out at a 95% confidence level; thus, a process parameter or their combination is considered significant if the *p*-value is lower than 0.05). Table 5 reports the ANOVA results, in which the significant parameters are highlighted by the bold text. While, Figure 7 and Figure 8 show the main effects plot and the significant interaction plot. In Figure 7 the significant terms (*E* and *C*) are highlighted by the continuous line.

As reported in Table 5, the results show that, the laser energy per scan line, the laser cleaning treatment and the interaction between them are statistically significant for the UTS. Among them, the single terms have a greater influence on the response variable if compared to their interaction, as highlighted by the contribution percentage which is greater than 22% against the 8% of the E*C term. This is also confirmed by the Fisher value, which is much greater than the tabulated ones for both *E* and *C*, i.e., ~12 and ~32 against 3.21 and 4.06 respectively. This is sufficient evidence to indicate that the single terms affect the ultimate tensile strength. In the main effects plot (see Figure 7), the significant parameters are highlighted using continuous lines. The figure shows that increasing the laser energy per scan line from 3 J/mm to 5 J/mm there is a decrease of the UTS to values of almost the half. The same trend is observed using or not the laser cleaning, highlighting in this way the efficacy of the treatment. In fact, by laser cleaning the surface of the sample, a rougher surface is obtained, therefore improving the joining between the materials. This is in accordance with the pertinent literature [17,42], in which it is stated that the combination of the surface activation without affection of the integrity of the laminate due to the cleaning of the surface leads to the strengthening of the joint. However, it is worth to note that using the highest power level, i.e., 200 W, involves the adoption of a higher scan speed in order to keep constant the laser energy. As a consequence, there is a shorter interaction time between the laser beam and the substrate, and therefore the heat does not diffuse into the inner layers and tends to be limited on the surface of the CFRP laminate. The result is a larger joined area that exceeds the threshold temperature required for joining. Moreover, in correspondence of the beam axis polycarbonate degradation takes place, while peripheral regions do not reach the melting temperature. In this way, the contribution because of these parts of the sample to the strength of the joint is very weak.

For sake of briefness and clearness, Figure 8 shows the interaction plot only for the significant interaction E*C. In particular, the greater the slope difference, the stronger the interaction. This is more evident in the first part of the plot, for lower values of *E*. This is ascribable to the fact that for lower values of laser energy the diffusion of the heat to the inner layers occurs without the degradation of the reinforcement, thus obtaining a more resistant joint.

It is important to highlight here that the results of the ANOVA test show the variability of the two-step laser joining process that contributes to about 30% to the total Seq.SS (see Table 5), thus conferring a certain degree of uncertainty to the experimental data processed. Moreover, the regression model is responsible for a systematic error between data and model results. Here the need of expert systems is able to consider both sources of discrepancy and propagate them to the experimental results, giving information on the level of uncertainty that in the proposed model is minimized by using genetic algorithms.

### Optimal Fuzzy Regression Model

The first step of the procedures was to find the best regression model able to fit the experimental data among the infinite possible combination of the input parameters and their powers. In particular, the number of terms were fixed at 6, according to the number of terms used in the ANOVA test.

The general model which can be drawn is reported in the following:

(5) $$\mathrm{UTS}=k_{1}\cdot P^{e_{1,1}}\cdot E^{e_{1,2}}\cdot C^{e_{1,3}}+k_{2}\cdot P^{e_{2,1}}\cdot E^{e_{2,2}}\cdot C^{e_{2,3}}+\cdots +k_{6}\cdot P^{e_{6,1}}\cdot E^{e_{6,2}}\cdot C^{e_{6,3}}.$$

In the latter equation, UTS is the response variable, *k*_1_ to *k*_6_ represent the empirical coefficients, evaluated by standard linear regression, while *e*_1,1_ to *e*_6,3_ the possible powers. Finally, *P*, *E*, *C* are the control factors. In particular, the first term *k*_1_ is the constant term, for which *e*_1,1_, *e*_1,2_, and *e*_1,3_ are equal to 0. While, the other powers were let to assume only few possible values, i.e., between −2 and 2 with a step of 0.5, with a total of nine possible values. In this way, the explored space is discrete and contains 9^5 × 3^ possible models, where 5 is the number of terms and 3 the number of variables constituting each term. In general, convergence was reached in less than 150 generations, in each of which 2000 individuals, i.e., models are evaluated. In practice, the genetic algorithm (GA) explores a space of cardinality *C* ≅ 2 × 10^14^ solving only 3 × 10^5^ models. Further 50 generations were computed to verify if mutation can move the optimum from a local minimum toward a better solution. The optimisation was run several times always obtaining the same result, ensuring in this way that the GA reached a global minimum.

It is worth noting that, despite the ANOVA does not indicate the power as statistically significant, in the Equation (5) the same has been maintained. This choice was made for two reasons: the first is that, in any case, the ANOVA shows a non-negligible error (about 30%). The second is because we wanted to test and stress the new procedure.

**Figure 9:**
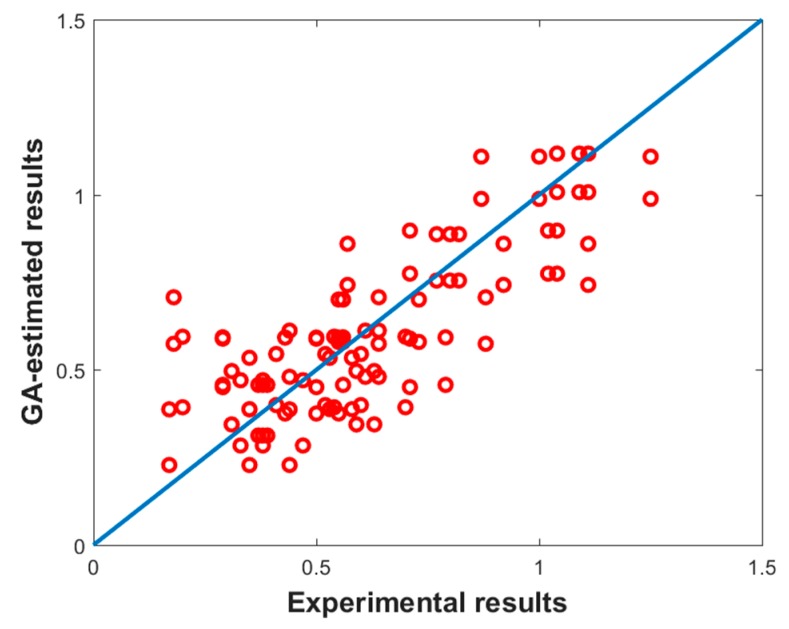
Comparison between GA-model and experimental results.

Table 6 reports the values of the coefficients and the powers of each term of the optimal regression model obtained by using the genetic algorithm presented in section computational, while Figure 9 shows the comparison among the model and the experimental results, which are characterized by a mean error of about 7%.

Starting from this empirical model, the fuzzy model is built considering the coefficients, *k*_1_ to *k*_6_ as triangular fuzzy numbers, denoted as $k_{1}^{*}$, to $k_{6}^{*}$. Thus, the resulting fuzzy model is:

(6) $${\mathrm{UTS}}^{*}=k_{1}^{*}+k_{2}^{*}\cdot \sqrt[{2}]{P^{3}}\cdot E^{2}\cdot C+k_{3}^{*}\cdot P^{2}\cdot C+k_{4}^{*}\cdot \frac{\sqrt{E}}{P^{2}\cdot \sqrt[{2}]{C^{3}}}+k_{5}^{*}\cdot \frac{1}{P\cdot C}+k_{6}^{*}\cdot E^{2}\cdot \sqrt{C}.$$

The aim was to produce a fuzzy input-output relation, based on the experimental observations, that links the control factors, i.e., laser energy, laser power and laser cleaning, to the achieved ultimate tensile strength. The model can be used to evaluate how much a given experimental sample, characterized by a certain value of *E*, *P* and *C*, and the corresponding UTS, belong to the fuzzy set defined by Equation (6). It is important to notice that the values of the process parameters are measured and deterministic thus remain regular numbers, while the uncertainty is modelled within the fuzzy coefficients.

Generally speaking, it is possible to state that the nominal model (Equation (5)) does not represent any experimental data (i.e., there is no experimental evaluation that can fall over the model surface). As the level of uncertainty is increased, measured by a decrease in the membership function, the model accommodates a larger number of samples with lower membership level. In other words, the fuzzy model is able to describe, as the membership function decreases, an increasing number of experimental data and, thanks to the genetic algorithm, with the highest degree of belonging to the fuzzy set defined by the model itself [29].

**Figure 10:**
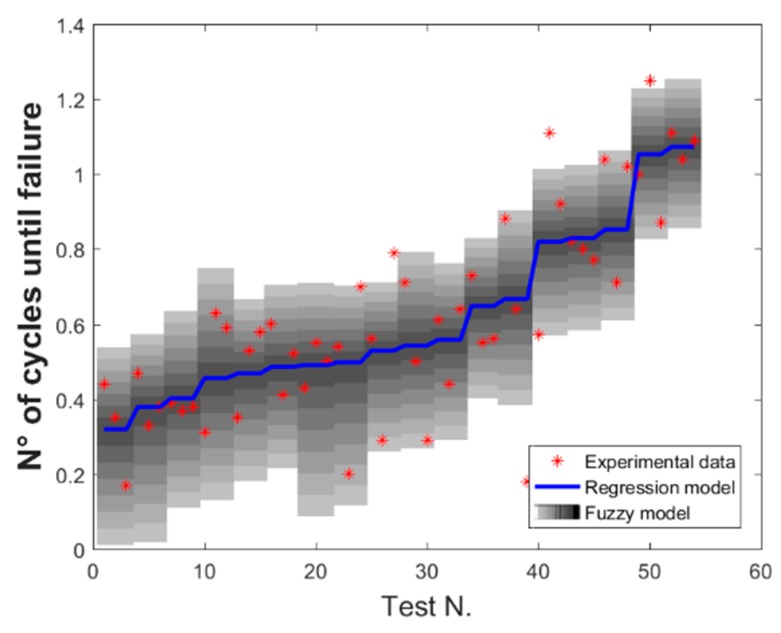
Fuzzy results for the ultimate tensile strength.

All the fuzzy parameters are described by eight α-cuts and the interval at each α-level is discretized with three points, the upper and the lower bound and a midpoint. For each α-cut, the transformation method requires, in a combinatorial scheme, the evaluation of the points at each α-level to the power of the number of fuzzy parameters, six in this case, leading to 729 evaluations. The transformation method requires that, for each α-cut, all these models are evaluated obtaining for each of them the hypersurface of the output quantity as a function of the process parameters. The fuzzy result for the given α-cut is then obtained by computing the envelope of these hypersurfaces, which are reported in Figure 10. In particular, along the x-axis are reported the experimental tests ordered for increasing values of the input parameters combinations, while along the y-axis the response variable, i.e., UTS.

From the inspection of the fuzzy results reported in Figure 9, the uncertainty level related to the fuzzy models appears to be not constant with respect to the parameters’ combination used during the experimental test. It is worth to note that the extent of the input uncertainty in the model, because of the choice of a specific confidence interval, is not only related to the accuracy of the regression model adopted but also to the variability of the process. So, the transformation method, which in this case was used to propagate the uncertainty to the outputs, also provides information about the uncertainty at the input level due to the regression model adopted. This effect can be therefore considered the reason for a non-constant level of uncertainty.

In general, this kind of process map can be used to select operational parameters in order to obtain a desired process output. They provide, as additional information, how much the uncertainty of the model and the process varies by changing the operational parameters. It is important to notice here that the variability of the process is highlighted by the combined fuzzy-genetic algorithm model through bands of uncertainty, represented in the latter figure by grey shaded areas. Moreover, it is worth to note that this information is not available by considering just the nominal regression model, nor directly obtained from the values of the confidence intervals.

**Figure 11:**
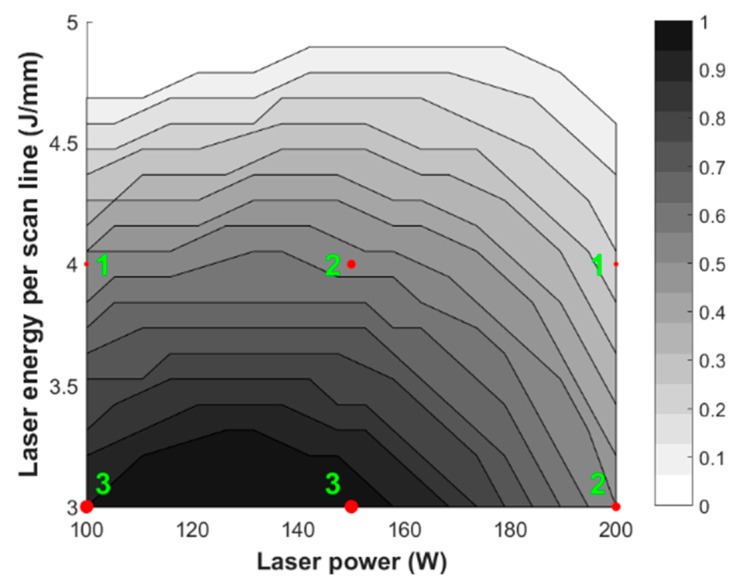
Fuzzy inverse map for the ultimate tensile strength.

The proposed model can also be inverted (see Figure 11) in order to obtain the most suitable operational parameters’ combinations, in terms of laser energy and laser power, while considering the laser cleaning as a constant, leading to a desired output. For the case study, the fuzzy-genetic algorithm model has been used to assess the optimal parameters in order to satisfy the highest resistance (set over 65% of the maximum value achieved, i.e., at about 0.8 kN). The membership level of the fuzzy model is represented as a grey shaded area (white to black corresponds to µ(x) from 0 to 1), while the experimental data and their occurrences as red dots (the dimension of each dot is proportional to the number of occurrences reported as green numbers).

From Figure 11, it is evident that there are different solutions that can satisfy the requested requirement of UTS > 0.8 kN, as highlighted by the grey shaded area. In particular, among the various combinations, the one with the lowest degree of uncertainty is given by the darkest area, which is characterized by a laser power lower than 150 W and a laser energy lower than 4 J/mm. This is also supported by the three experimental occurrences over three repetitions, reported as green numbers, which fall exactly in the darkest area. Moreover, analysing the fuzzy inverse map it is recommended to use a combination of *E* and *P* within 3–3.3 J/mm and 100–160 W, respectively, as highlighted by the darkest area. Among these, wanting to remain in the central part of the optimal working (i.e., the darkest area) area and therefore the farthest from the lighter zones, reasonable values for *E* and *P* may be 3.15 J/mm and 135 W, respectively. It is worth to highlight that this does not mean that it is not possible to satisfy the requirement with the other combinations, but that those scenarios are characterized by a certain degree of uncertainty, which is higher for a high value of laser power and laser energy because the fuzzy map is lighter.

## 4. Conclusions

This study presents an innovative two-step laser joining process for the realization of composite/polymer hybrid structures by previously laser cleaning the composite surface. The main target was to investigate the efficacy of the pre-treatment on the improvement of the joint strength. Then, once demonstrated its technological validity, a fuzzy-genetic algorithm model has been developed and successfully applied for the optimisation of the full process, in terms of the best input parameters’ combination able to guarantee the highest mechanical performances.

The experimental campaign, based on design of experiment, has shown that the laser cleaning pre-treatment enhance the joining between the two materials because of the removal of the first matrix layer of the epoxy resin. This leads to a more homogeneous distribution of the heat at the interface layer and an increase of the CFRP roughness that allows (enhance) the mechanical bonding between the PC and CFRP. Both the effects ensuring an effective joining between CFRP and PC without any degradation. Among the different scenarios investigated in this research work, the most effective appeared to be a laser energy per scan line of 3 mJ and a laser power of 200 W with the pre-treatment of the surface.

The computational part regarded the development of a fuzzy model starting from the experimental findings. In particular, both the nominal empirical model and the support of the fuzzy numbers, considered as triangular fuzzy numbers, are evaluated by using a genetic-based optimisation algorithm. While, the transformation method was used to handle uncertainty propagation of the input parameters, i.e., laser energy, laser power and laser cleaning, to the output variable, i.e., ultimate tensile strength. Therefore, the proposed combined fuzzy-genetic algorithm model is aimed at selecting the laser parameters in order to satisfy the highest resistance, providing, as additional information, how much the uncertainty of the model and the process varies by changing those operational parameters.

The genetic algorithm is able to reproduce the experimental data with an error of about 7%, lower than the error associated with the unpredictable factors in the ANOVA test of about 30%. In fact, the variability of the process is highlighted by the fuzzy model through the bands of uncertainty. It is important to notice that this information is not available by considering just the nominal regression model, nor directly obtained from the values of the confidence interval. Finally, in order to guarantee the highest strength of the joint the fuzzy inverse map suggests that the best combination is given by a laser power within the range of 100–160 W and a laser energy per scan line of 3–3.3 J/mm, after laser cleaning the surface of the CFRP laminate.

## Figures and Tables

**Table 1:** Main mechanical and thermal properties of the adopted materials.

Properties	Units	Material	
		CFRP	PC
Young’s Modulus	GPa	175.3	2.4
Ultimate Tensile Strength	MPa	962.7	65
Melting Temperature	°C	–	230
Glass Transition Temperature	°C	165	154
Degradation Temperature	°C	450	540

**Table 2:** Processing conditions for the laser cleaning treatment.

Laser Parameters	Units	Values
Wavelength	nm	1064
Average Power	W	30
Pulse Frequency	kHz	30
Pulse Duration	ns	50
Laser Scan Speed	mm/s	2000
Hatch Distance	µm	40
Strategy	–	Line

**Table 3:** Diode laser characteristics for the joining process.

Laser Parameters	Units	Values
Wavelength	nm	975
Peak Power	W	200
Beam Profile	–	Circular
Beam Diameter	mm	6
Beam Quality	mm·mrad	22

**Table 4:** Multilevel factorial plan: 3 terms of *P* × 3 terms of *E* × 2 terms of *C* = 18 experimental scenarios.

Control Factors	Symbol	Units	Levels		
Laser Power	*P*	W	100	150	200
Laser Energy per scan line	*E*	J/mm	3	4	5
Laser Cleaning	C	–	Yes		No

**Table 5:** ANOVA table for the ultimate tensile strength.

Source	DoF	Seq.SS	Π%	Adj.SS	Adj.MS	F-Value	*p*-Value
*P*, W	2	0.04032	1.09%	0.03240	0.01620	0.51	0.603
***E*** **, J/mm**	**2**	**0.82507**	**22.24%**	**0.80534**	**0.40267**	**12.72**	**0.000**
***C***	**1**	**1.07131**	**28.88%**	**1.03587**	**1.03587**	**32.73**	**0.000**
*P* × *E*,	4	0.06974	1.88%	0.07406	0.01852	0.58	0.675
*P* × *C*	2	0.17085	4.60%	0.15934	0.07967	2.52	0.094
***E* × *C***	**2**	**0.29832**	**8.04%**	**0.29832**	**0.14916**	**4.71**	**0.015**
Error	39	1.23447	33.27%	1.23447	0.03165		
Lack-of-Fit	4	0.13320	3.59%	0.13320	0.03330	1.06	0.392
Pure Error	35	1.10127	29.68%	1.10127	0.03146		
Total	52	3.71008	100.00%				

**Table 6:** Coefficients and powers of the terms of the optimal regression model.

Term (*i*)	$\mathrm{Coefficient}\;k_{i}$	Power of *P*	Power of *E*	Power of *C*
1	2.88	0	0	0
2	5.47 × 10^−6^	1.5	2	1
3	−1.45 × 10^−5^	2	0	1
4	1.18 × 10^−4^	−2	0.5	−1.5
5	−419.56	−1	0	−1
6	−0.04	0	2	0.5
